# Efficacy and Safety of IncobotulinumtoxinA for the Treatment of Blepharospasm: A Multicenter, Phase 3 Study in Japan

**DOI:** 10.3390/toxins18020109

**Published:** 2026-02-20

**Authors:** Toshiaki Goseki, Yoshihito Mochizuki, Akiko Masuda, Motohiro Kiyosawa, Ryosuke Miyamoto, Masato Wakakura, Akiko Yamagami, Yohei Mukai, Akihiro Shinkai, Mutsumi Iijima, Kousuke Baba, Hideki Chuman, Masahiro Hashizuka, Shohei Tateishi, Akiko Kimura

**Affiliations:** 1 Department of Ophthalmology, International University of Health and Welfare, Atami Hospital, Atami 413-0012, Japan; 2 Department of Ophthalmology, Kitasato University, Sagamihara 252-0375, Japan; 3 Department of Ophthalmology, Hyogo Medical University, Nishinomiya 663-8501, Japan; 4 Jiyugaoka Kiyosawa Eye Clinic, Tokyo 152-0035, Japan; 5 Department of Neurology, Tokushima University Hospital, Tokushima 770-8503, Japan; 6 Department of Ophthalmology, Inouye Eye Hospital, Tokyo 101-0062, Japan; 7 Department of Neurology, National Center of Neurology and Psychiatry, Tokyo 187-8551, Japan; 8 Department of Ophthalmology, Faculty of Medicine, Graduate School of Medicine, Hokkaido University, Sapporo 060-8648, Japan; 9 Department of Neurology, School of Medicine, Tokyo Women’s Medical University, Tokyo 162-8666, Japan; 10 Department of Neurology, Graduate School of Medicine, The University of Osaka, Osaka 565-0871, Japan; 11 Department of Ophthalmology, Faculty of Medicine, University of Miyazaki, Miyazaki 889-1692, Japan; 12 Clinical Development Department, Teijin Pharma Limited, Tokyo 100-8585, Japan; 13 Clinical Development Control Department, Teijin Pharma Limited, Tokyo 100-8585, Japan; 14 Kind Eye Clinic, Kobe 650-0022, Japan

**Keywords:** blepharospasm, botulinum toxin, dystonia, East Asia, efficacy, incobotulinumtoxinA, Japan, phase 3 study, safety, Jankovic Rating Scale

## Abstract

This open-label, uncontrolled, single-arm, multicenter, phase 3 study evaluated the efficacy and safety of incobotulinumtoxinA in Japanese patients with blepharospasm. Eligible patients received incobotulinumtoxinA injections at fixed doses (50, 75, or 100 units [U] for those who had previously received botulinum toxin treatment; 50 U for treatment-naïve patients), followed by flexible doses up to 100 U for 48 weeks, with at least 6-week intervals. In total, 29 Japanese patients were enrolled (26 [89.7%] women, mean age 64.6 years, mean baseline Jankovic Rating Scale [JRS] severity score 3.24). The primary endpoint, the least squares mean of change in JRS severity scores from baseline to 6 weeks after the first injection, was −2.08 (95% confidence interval: −2.49, −1.66), meeting the prespecified efficacy criteria. The secondary endpoint results (JRS severity, frequency, and total scores for 48 weeks; Blepharospasm Disability Index; Patient Evaluation of Global Response; and fast blinking test) supported the efficacy of repeated incobotulinumtoxinA injections. Adverse events (AEs) occurred in 19 (65.5%) patients, with eyelid ptosis being the most common treatment-related AE (4 [13.8%] patients). No severe or serious AEs were reported. IncobotulinumtoxinA demonstrated sustained efficacy in Japanese patients with blepharospasm, without new safety concerns. (Japan Registry of Clinical Trials identifier, jRCT2031230711)

## 1. Introduction

Blepharospasm is a subtype of dystonia characterized by bilateral, synchronous, and involuntary contractions of the orbicularis oculi muscle, the etiology of which has not been well established [1]. Patients with blepharospasm commonly experience motor or non-motor ocular symptoms such as increased blinking, photophobia, dry eyes, and ocular pain [2,3]. Patients may also develop non-motor, extraocular symptoms, such as psychiatric, mood, and cognitive disorders [2,3,4,5], or they may exhibit sleep problems that impair their quality of life [4,6]. The prevalence of blepharospasm is estimated to be 4.24 per 100,000 people and is higher in women (4.78 per 100,000) than in men (3.08 per 100,000) [7]. According to a 2023 survey conducted by the Ministry of Health, Labour and Welfare, the number of patients with blepharospasm in Japan is estimated at approximately 28,000 [8].

The first-line treatment for blepharospasm recommended in many countries is botulinum toxin [5,9,10,11] and in Japan, onabotulinumtoxinA was the first formulation available for clinical use [12]. Treatment options other than botulinum toxin include supportive treatments such as oral pharmacotherapy, photochromatic modulation using lens tints, and surgical interventions [1]. IncobotulinumtoxinA is a highly purified botulinum toxin type A product [13]. It is designed to contain a 150-kDa neurotoxin as the active component with unnecessary clostridial proteins removed, and is characterized by low immunogenicity [13,14]. IncobotulinumtoxinA has been demonstrated to be therapeutically equivalent to 900-kDa onabotulinumtoxinA for treating blepharospasm, with a conversion ratio of 1:1 [15].

IncobotulinumtoxinA is already approved for the treatment of blepharospasm in many countries worldwide [16] (for blepharospasm and hemifacial spasm in adults in major European countries [17]). In Japan, as of October 2025, it is available for clinical use in upper- and lower-limb spasticity and chronic sialorrhea [18]. The efficacy and safety of incobotulinumtoxinA in patients with blepharospasm have been demonstrated in an active comparator (onabotulinumtoxinA)-controlled phase 3 study [19], a placebo-controlled phase 3 study in patients who had previously received botulinum toxin type A treatment [20], and a placebo-controlled phase 3 study in botulinum toxin-naïve patients [21]. However, data on East Asian populations remain limited. This phase 3 trial (Japan Registry of Clinical Trials identifier, jRCT2031230711) assessed the efficacy and safety of incobotulinumtoxinA in Japanese patients with blepharospasm.

## 2. Results

### 2.1. Patient Disposition

**Figure 1:**
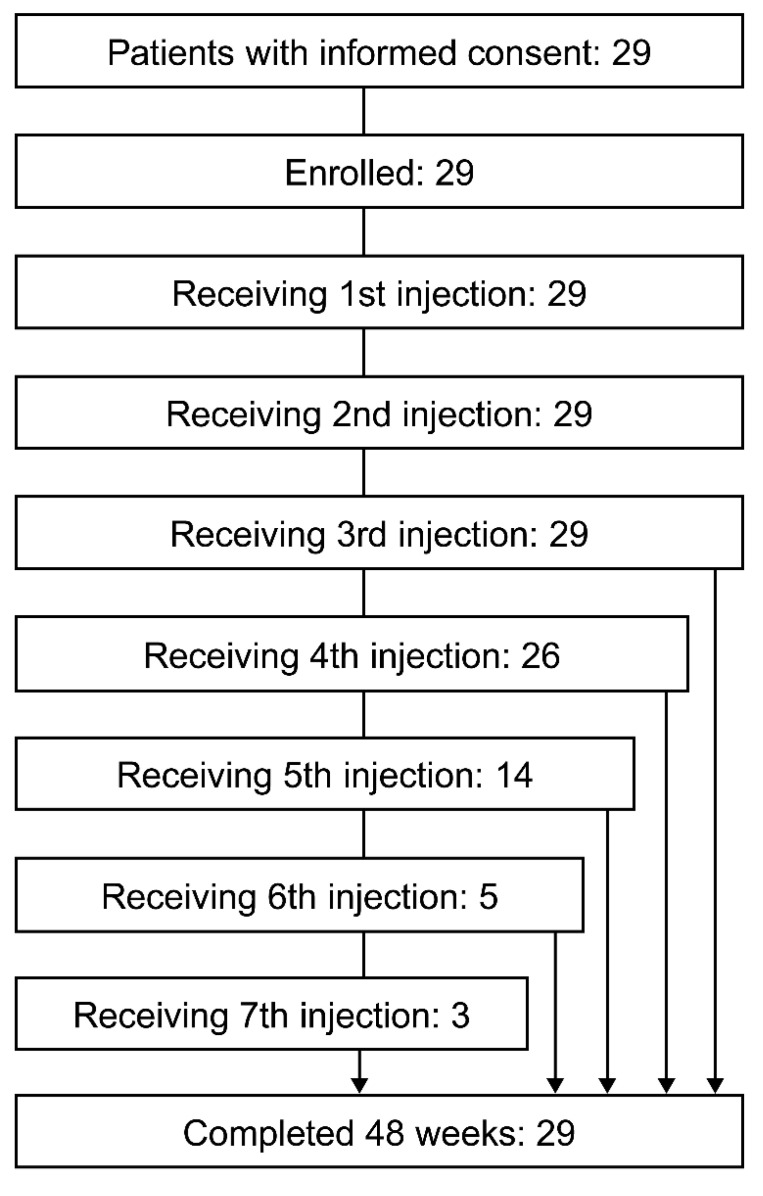
Patient disposition.

A total of 29 patients provided written informed consent and were enrolled in the study at 14 institutions across Japan (study period: 23 April 2024 to 10 July 2025). None of them discontinued the study. Among these 29 patients, 26 received a fourth injection and 3 received a seventh injection (Figure 1). All 29 patients were included in the full analysis set (FAS) and in the safety population.

### 2.2. Baseline Characteristics

All 29 patients were Japanese (Table 1). The majority (26 [89.7%]) of the patients were women, and the mean ± standard deviation (SD) age was 64.6 ± 11.18 years. The mean ± SD duration of blepharospasm was 86.55 ± 87.39 months. Among the 29 patients, 23 (79.3%) had previously received botulinum toxin treatment for blepharospasm (Table 1), with all 23 receiving onabotulinumtoxinA. The details of the last 2 doses of onabotulinumtoxinA are summarized in Table S1. The mean ± SD Jankovic Rating Scale (JRS) severity score [22,23] at baseline was 3.24 ± 0.58 (Table 1). In total, 6 (20.7%) patients had a medical history, and the most common (≥2 patients) medical history was cataract and eyelid ptosis (2 patients each). A total of 27 (93.1%) patients had comorbidities (Table 1), and the most common comorbidity was hypertension, which was reported in 8 (27.6%) patients. One (3.4%) patient had symptoms of blepharoptosis at baseline.

### 2.3. IncobotulinumtoxinA Dosing Status

The total dose for the first injection cycle of incobotulinumtoxinA was 50 units (U) in 16 (55.2%) patients, 75 U in 9 (31.0%) patients, and 100 U in 4 (13.8%) patients. The mean ± SD dose for the first injection cycle was 64.66 ± 18.319 U in all patients, with 50 ± 0.0 U in botulinum toxin-naïve patients and 68.48 ± 18.795 U in patients who had previously received botulinum toxin treatment (Table S2). The most common dosing intervals between the first and second injection cycles were ≥8 to <12 weeks and ≥12 to <16 weeks, both observed in 10 (34.5%) patients each (Table S3). The dose data for the first injection cycle by affected muscle are summarized in Table S4. The total number of injection sites for the first injection cycle in 29 patients is provided in Figure S1.

### 2.4. Primary Endpoint

The least squares (LS) mean of change in the JRS severity score from baseline to 6 weeks after the first injection was −2.08 (95% confidence interval [CI]: −2.49, −1.66; mixed-effects model for repeated measures [MMRM] analysis). The results met the prespecified efficacy criteria (the upper limit of the 95% CI was below the threshold value of −0.59) (Table 2). Similar results were obtained in the sensitivity analysis.

### 2.5. Efficacy of IncobotulinumtoxinA Stratified by Patient Characteristics

**Figure 2:**
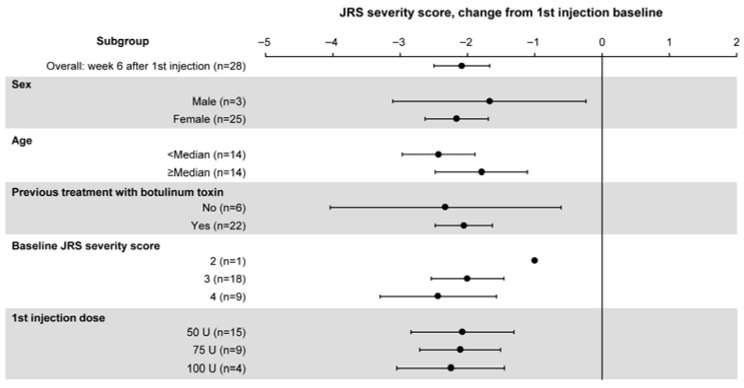
Change from baseline to 6 weeks after the first injection in the JRS severity score by subgroup (FAS). Results are presented as mean (dots) and 95% CI (error bars). The analysis for 6 weeks after the first injection was performed with 28 patients, and 1 patient who missed the scheduled visit was excluded from the analysis. CI, confidence interval; FAS, full analysis set; JRS, Jankovic Rating Scale.

Subgroup analyses indicated an improvement in the JRS severity score at 6 weeks after the first incobotulinumtoxinA injection, regardless of sex, age, a history of botulinum toxin treatment, baseline JRS severity score, or first injection dose (Figure 2).

### 2.6. Secondary Endpoints

**Figure 3:**
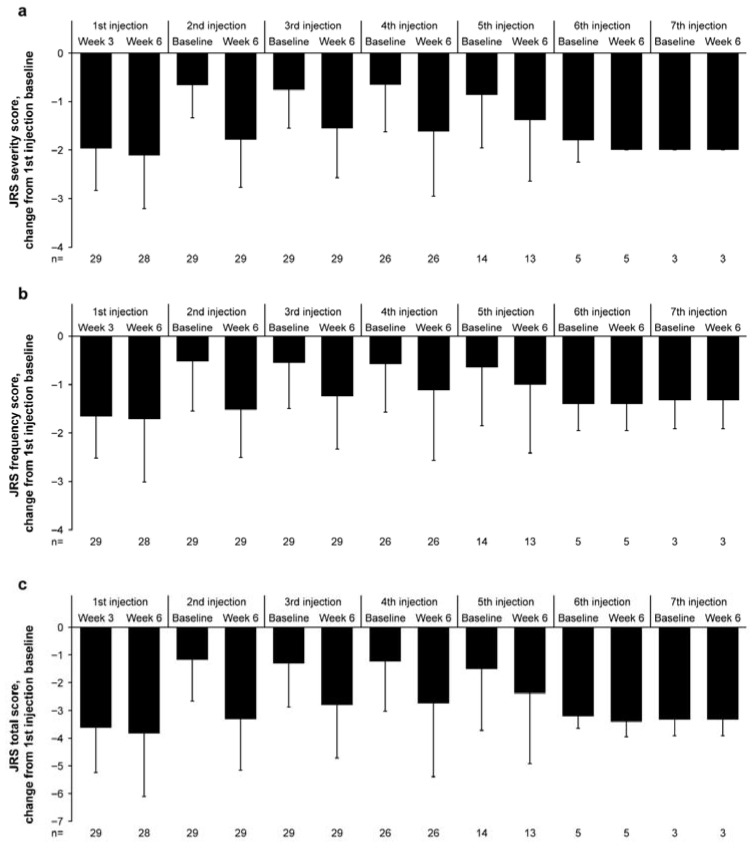
Change from baseline in (**a**) JRS severity, (**b**) JRS frequency, and (**c**) JRS total scores (FAS). Results are presented as mean and SD. The analysis for 6 weeks after the first injection was performed with 28 patients, and 1 patient who missed the scheduled visit was excluded from the analysis. FAS, full analysis set; JRS, Jankovic Rating Scale; SD, standard deviation.

At baseline (before the initiation of the first injection), the mean ± SD of the JRS severity score, JRS frequency score, and JRS total score was 3.24 ± 0.58, 2.72 ± 0.75, and 5.97 ± 1.21, respectively (*n* = 29 for all; Table 1). The JRS severity score (Figure 3a), frequency score (Figure 3b), and total score (Figure 3c) exhibited a trend toward reduction from baseline up to 48 weeks with repeated incobotulinumtoxinA injections.

The mean ± SD of change in the JRS severity score from baseline was −1.97 ± 0.865 at 3 weeks after the first injection (*n* = 29) and −2.11 ± 1.100 at 6 weeks after the first injection (*n* = 28). During subsequent injections, the change in the JRS severity score at 6 weeks (mean ± SD) ranged from −1.38 ± 1.261 to −2.00 ± 0.00. The mean ± SD of change from baseline in the JRS frequency score was −1.66 ± 0.857 at 3 weeks after the first injection and −1.71 ± 1.301 at 6 weeks after the first injection. At subsequent injections, the change in the JRS frequency score at 6 weeks (mean ± SD) ranged from −1.00 ± 1.414 to −1.52 ± 0.986. The mean change from baseline in the JRS total score was −3.62 ± 1.613 at 3 weeks after the first injection and −3.82 ± 2.278 at 6 weeks after the first injection. During subsequent injections, the change in the JRS total score at 6 weeks (mean ± SD) ranged from −2.38 ± 2.534 to −3.40 ± 0.548. The JRS severity, frequency, and total scores showed a reduction (improvement) from the first injection baseline at all assessment points, indicating sustained efficacy of incobotulinumtoxinA with a maximum of 7 injection cycles.

**Figure 4:**
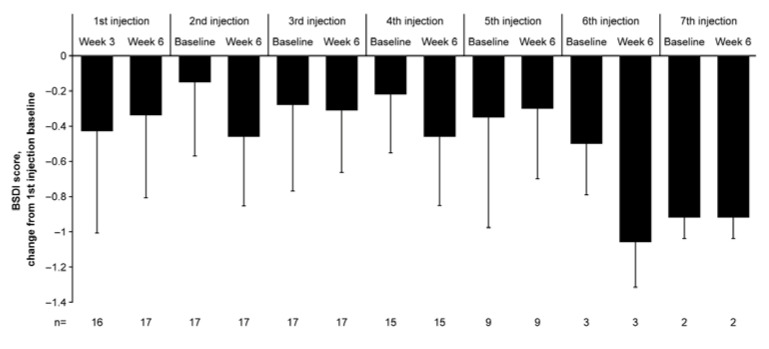
Change from baseline in the BSDI score (FAS). Results are presented as mean and SD. BSDI, Blepharospasm Disability Index; FAS, full analysis set; SD, standard deviation.

The mean ± SD Blepharospasm Disability Index (BSDI) [19,23] score at baseline was 1.65 ± 0.90 (*n* = 17, Table 1). The BSDI score showed a sustained decrease from baseline to 48 weeks with repeated incobotulinumtoxinA injections (Figure 4). The mean ± SD of change from baseline in the BSDI score was −0.43 ± 0.577 at 3 weeks after the first injection (*n* = 16) and −0.34 ± 0.466 at 6 weeks after the first injection (*n* = 17). During subsequent injections, the change in the BSDI score (mean ± SD) ranged from −0.15 ± 0.420 to −0.92 ± 0.118 at baseline and from −0.30 ± 0.398 to −1.06 ± 0.255 at 6 weeks. The BSDI score showed a reduction (improvement) from the first injection baseline at all assessment points.

The mean ± SD Patient Evaluation of Global Response (PEGR) [23,24] score was 2.0 ± 1.13 for the first injection cycle (*n* = 29). The PEGR score showed a trend toward a reduction (improvement) from the first injection baseline in patient-assessed blepharospasm symptoms throughout the study period (Table S5). The score in the fast blinking test (mean ± SD) was 2.3 ± 0.8 at baseline (*n* = 29), 1.0 ± 0.9 at 3 weeks after the first injection (*n* = 29), and 0.7 ± 0.7 at 6 weeks after the first injection (*n* = 28). During subsequent injections, the fast blinking test score (mean ± SD) ranged from 1.0 ± 0.0 to 1.7 ± 1.0 at baseline and 0.9 ± 0.8 to 1.2 ± 0.9 at 6 weeks. The quality of blinking showed improvement from the first injection baseline at all assessment points with repeated incobotulinumtoxinA injections (Figure S2).

### 2.7. Safety

Throughout the study period, adverse events (AEs) and treatment-related AEs were observed in 19 (65.5%) and 7 (24.1%) patients, respectively, in the safety population (Table 3). None of the patients reported serious AEs, serious treatment-related AEs, or AEs leading to death or study discontinuation, and all AEs were mild to moderate in severity. The most commonly reported AEs were nasopharyngitis (5 [17.2%] patients) and eyelid ptosis (4 [13.8%] patients). The most commonly reported treatment-related AE was eyelid ptosis, which was observed in 4 (13.8%) patients (Table 3). All patients reporting treatment-related AEs recovered without sequelae and continued treatment with incobotulinumtoxinA.

The proportion of patients experiencing AEs decreased with the number of injections, from 37.9% during the first injection cycle to 0.0% during the seventh injection cycle (Table S6). No notable trends were observed in the incidence rates of AEs when patients were stratified by incobotulinumtoxinA dose (58.8% [10/17 patients] at ≤50 U, 50.0% [7/14 patients] at >50 to ≤75 U, and 44.4% [4/9 patients] at >75 to ≤100 U) or dosing interval (36.4% [4/11 patients] at ≥6 to <8 weeks, 38.1% [8/21 patients] at ≥8 to <12 weeks, and 30.0% [6/20 patients] at ≥12 weeks).

Among the AEs of special interest (effects on distal muscles, hypersensitivity reactions, eye disorders, convulsive attacks, and injection site reactions), eye disorders (31.0% [9/29 patients]) and effects on distal muscles (24.1% [7/29 patients]) were the most commonly observed (Table S7). There were no notable findings related to laboratory parameters or vital signs.

## 3. Discussion

More than two decades have passed since incobotulinumtoxinA was first approved for the treatment of blepharospasm in Europe and the United States [16]. Nevertheless, limited clinical data were available for East Asian patient populations until now. This phase 3 study assessed the efficacy and safety of incobotulinumtoxinA in Japanese patients with blepharospasm. The primary endpoint, LS mean of change from baseline in the JRS severity score to 6 weeks after the first injection, met the prespecified efficacy criteria. Overall, the results indicated sustained efficacy of repeated incobotulinumtoxinA injections for up to 48 weeks using both clinician-rated scales (JRS severity, frequency, and total scores and fast blinking test) and patient-reported outcomes (BSDI and PEGR). None of the patients discontinued incobotulinumtoxinA due to a lack of efficacy, which may support the low immunogenicity [14] and sustained efficacy of this botulinum toxin formulation. Graphical abstracts (graphical plain language summary in English and Japanese) are presented as Figures S3 and S4.

There were no notable differences in baseline patient characteristics compared to the existing placebo-controlled phase 3 studies, except for a numerically higher proportion of female patients (89.7% in the current study vs. 59–65% in previous studies) [20,21]. The mean JRS severity score at baseline was not notably different across studies (3.24 in the current study vs. 2.94–3.12 in a previous study) [20]. The LS mean of change in the JRS severity score from baseline to 6 weeks after the first injection (−2.08) showed a clinically meaningful change (>2-point improvement in the JRS total score, equivalent to >1-point improvement in the JRS severity score) [23]. The JRS severity, frequency, and total scores showed sustained reductions with repeated doses of incobotulinumtoxinA, consistent with the findings of previous phase 3 studies [20,21]. Similarly, BSDI and PEGR results support the efficacy of incobotulinumtoxinA, which is in line with the existing phase 3 studies [20,21]. As demonstrated in previous phase 3 studies [20,21], our subgroup analyses indicated the efficacy of incobotulinumtoxinA, irrespective of a history of botulinum toxin treatment. Furthermore, the current efficacy results showed a trend toward improvement in the JRS, BSDI, and fast blinking test at each injection baseline, although no statistical comparison with the first injection baseline was performed. These data suggest a cumulative effect of repeated incobotulinumtoxinA injections, as shown in previous clinical trials [25,26].

Eyelid ptosis was observed as AEs and treatment-related AEs in 4 (13.8%) patients, which is attributable to the diffusion of incobotulinumtoxinA into the levator palpebrae superioris muscle that resulted in eyelid muscle relaxation. There were no AEs resulting in death, serious AEs, AEs leading to study discontinuation, or severe AEs. There was no trend toward an increase in the incidence rate of AEs with repeated incobotulinumtoxinA injections. Furthermore, the AE incidence rate did not show an increase in patients receiving incobotulinumtoxinA at short (<8 weeks, minimum 6 weeks) dosing intervals or at high doses. The most common AEs of special interest were eye disorders and effects on distal muscles. Overall, the safety results are largely consistent with the findings of previous clinical trials [20,21] and indicate that incobotulinumtoxinA was well tolerated without any new safety concerns.

This Japanese phase 3 study was designed to allow incobotulinumtoxinA injections at a maximum dose of 100 U and the shortest interval of 6 weeks in view of its low immunogenicity [14]. A conversion ratio of 1:1 has been previously established between onabotulinumtoxinA and incobotulinumtoxinA [15]. Thus, it was considered that for patients with a history of botulinum toxin treatment, those who did not achieve sufficient therapeutic effects with the indicated dosage and administration in Japan (a maximum dose of 45 U and the shortest interval of 8 weeks) [12] were enrolled in this study with the expectation of effects exceeding those of the previous treatment. Our safety data did not indicate an increase in the incidence rates of AEs in patients who received incobotulinumtoxinA with dosing intervals of <8 weeks or doses exceeding 45 U. Taken together, these Japanese phase 3 data support the efficacy of incobotulinumtoxinA administered at flexible intervals and doses without safety concerns.

This phase 3 study is the first to demonstrate the efficacy and safety of incobotulinumtoxinA in East Asian patients with blepharospasm. The use of the JRS severity score as the primary endpoint measure is consistent with that in other placebo-controlled phase 3 studies on incobotulinumtoxinA [20,21]. The use of multiple outcome measures, consisting of both clinician-rated and patient-reported scales, enabled a comprehensive evaluation of treatment efficacy. However, this study has some limitations. First, the single-arm design makes it difficult to directly compare our results with those of the existing placebo-controlled phase 3 studies [20,21]. Second, the observation period was limited to 48 weeks, necessitating assessment of the long-term safety and effectiveness of incobotulinumtoxinA in East Asian patients treated in real-world clinical settings. Lastly, although the sample size required for the primary efficacy analysis was achieved, the number of patients was limited with regard to the characteristics analyzed in the subgroups. Future research should focus on accumulating evidence for incobotulinumtoxinA treatment in East Asian patients with blepharospasm having diverse backgrounds through larger studies.

## 4. Conclusions

This study demonstrated the sustained efficacy of incobotulinumtoxinA at a maximum dose of 100 U in Japanese patients with blepharospasm without new safety concerns. After initiating incobotulinumtoxinA treatment in patients with blepharospasm, an improvement in symptoms was observed that continued for 48 weeks with repeated injections. These results are consistent with those of global phase 3 studies and support the inclusion of incobotulinumtoxinA as a well-tolerated treatment option in Japanese clinical practice.

## 5. Materials and Methods

### 5.1. Study Design and Overview

This open-label, uncontrolled, single-arm, phase 3 clinical trial (Japan Registry of Clinical Trials identifier, jRCT2031230711, date of registration: 15 March 2024) was conducted at 14 participating institutions across Japan from 23 April 2024 to 10 July 2025 (up to 48 weeks).

**Figure 5:**
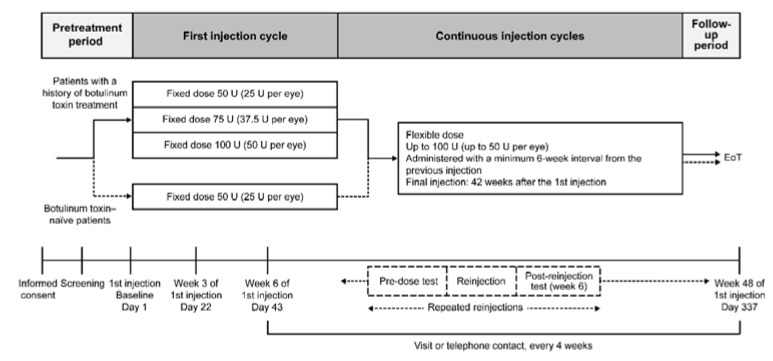
Trial design. EoT, end of trial; U, unit.

Patients with blepharospasm were enrolled and received incobotulinumtoxinA injections into the affected muscles at a fixed total dose, followed by repeated flexible doses at intervals of at least 6 weeks (Figure 5). The dosage of incobotulinumtoxinA (fixed dose of 50, 75, or 100 U for those who had previously received botulinum toxin treatment, 50 U for botulinum toxin-naïve patients; flexible dose up to 100 U) was determined based on the regimens used in previous phase 3 trials [20,21]. The incobotulinumtoxinA dose for individual patients at the first injection cycle was determined by the investigator based on their blepharospasm symptoms and dose regimen for previous botulinum toxin treatment. The incobotulinumtoxinA dose and dosing interval for individual patients at the second and subsequent injection cycles were determined by the investigator based on the patients’ blepharospasm symptoms, including treatment response and the duration of effect. The selection of muscles and the number of injection sites were also determined by the investigator based on the patients’ blepharospasm symptoms.

The study was approved by the Institutional Review Board of each participating institution.

### 5.2. Patient Inclusion and Exclusion Criteria

Patients aged 18–80 years with a clinical diagnosis of blepharospasm and a JRS severity score of ≥2 were eligible for participation in the study. Patients with previous botulinum toxin treatment for blepharospasm were eligible for participation if they had recurrent symptoms of blepharospasm that required botulinum toxin treatment and a minimum 10-week interval between the last treatment and baseline assessment. The key exclusion criteria were drug-induced blepharospasm due to psychotropic drugs, apraxia of eyelid opening as the predominant symptom, no response to previous botulinum toxin treatment for blepharospasm, previous botulinum toxin treatment for purposes other than blepharospasm (e.g., hemifacial spasm, cervical dystonia, spasticity or aesthetic use) within 16 weeks before the baseline assessment, or previous surgical treatment for dystonia or periorbital myectomy. Patients who initiated or discontinued any drug for the treatment of dystonia, including blepharospasm, or changed the dosage within 12 weeks prior to the assessment were excluded. Patients with systemic neuromuscular junction disorders (e.g., myasthenia gravis and Lambert-Eaton myasthenic syndrome) or amyotrophic lateral sclerosis were also excluded.

### 5.3. Treatment

IncobotulinumtoxinA (Merz Therapeutics GmbH, Frankfurt am Main, Germany; 100 U per vial; Table S8) was administered by investigators who had completed the training for study conduct, drug administration, and assessments required for this clinical trial. The first injection was administered as a fixed total dose split between the affected muscles (e.g., orbicularis oculi, corrugator supercili, procerus, nasalis, levator labii superioris alaeque nasi, and frontalis muscle). For subsequent injections, flexible total doses of incobotulinumtoxinA were administered to the affected muscles. Subsequent injection cycles were performed at intervals of at least 6 weeks, and the last administration of incobotulinumtoxinA was set at 42 weeks after the first injection cycle.

### 5.4. Endpoints

The primary endpoint was the LS mean of change in the JRS severity score from baseline to 6 weeks after the first injection of incobotulinumtoxinA (MMRM analysis). Treatment with incobotulinumtoxinA was judged to be efficacious when the upper limit of the 95% CI of the primary endpoint was below the threshold of −0.59. This threshold was defined based on the maximum treatment effect with placebo at 6 weeks after the first injection assumed from the results of a previous phase 3 trial [21].

The secondary endpoints included JRS severity, frequency, and total scores [27] assessed up to 48 weeks; BSDI score [27]; PEGR score; and results of the fast blinking test. Safety was primarily assessed based on the incidence rate of AEs and treatment-related AEs over 48 weeks. The effects on the distal muscles, hypersensitivity reactions, eye disorders, convulsive attacks, and injection site reactions were recorded as AEs of special interest.

### 5.5. Assessments

The JRS [22,23] was administered by trained investigators (JRS contact information and permission to use: Mapi Research Trust, Lyon, France, https://eprovide.mapi-trust.org [accessed on 10 October 2025]). The BSDI [19,20] and PEGR [24] were self-administered by patients throughout the study period. A fast blinking test [3] was performed in accordance with the Japanese guidelines for blepharospasm [5] (Table S8). AEs and treatment-related AEs were coded using the Medical Dictionary for Regulatory Activities (MedDRA) version 27.1 and summarized by system organ class (SOC) and preferred term (PT). AEs for which a causal relationship with incobotulinumtoxinA could not be ruled out were considered treatment-related AEs. AEs and treatment-related AEs were recorded based on the diagnosis established by the attending physician or signs and symptoms observed in each patient.

### 5.6. Statistical Analyses

The target sample size was 25 patients. The threshold for the primary endpoint was set to −0.59, and the expected value for the primary endpoint was estimated at −1.38 based on the LS mean of change from baseline to 3 or 6 weeks (MMRM analysis) after the first injection observed with incobotulinumtoxinA in previous phase 3 trials [19,20,21]. Assuming an SD of 1.269 for the change from the baseline, a one-sided significance level of 2.5%, and a power of 80% for a one-sample *t*-test, a sample size of 23 patients was estimated. Considering patient discontinuation, the final sample size was calculated as 25 patients.

Among patients who received at least 1 dose of incobotulinumtoxinA injection, those who provided at least 1 data point for efficacy assessments were included in the FAS. Similarly, patients who provided at least 1 data point for safety assessment were included in the safety population.

The primary endpoint was analyzed in the FAS using an MMRM model that included the change in the JRS severity score from baseline as an objective variable, baseline JRS severity score as an explanatory variable, and time point as a fixed effect. The LS mean and 95% CI at 6 weeks after the first injection were calculated. In the sensitivity analysis, descriptive statistics and 95% CI based on a one-sample *t*-test were calculated. All other efficacy and safety results are summarized as mean ± SD for continuous variables and the number and proportion of patients for categorical variables.

Missing values were not imputed, and multiplicity was not adjusted. All statistical analyses were performed using SAS version 9.4 (SAS Institute, Cary, NC, USA).

## Figures and Tables

**Table 1:** Baseline demographics and patient characteristics (FAS).

Characteristic	FAS (*n* = 29)
Japanese, *n* (%)	29 (100.0)
Sex, *n* (%)	
Female	26 (89.7)
Male	3 (10.3)
Mean ± SD age, years	64.6 ± 11.18
Mean ± SD BMI, kg/m^2^	22.78 ± 4.48
Mean ± SD disease duration, months	86.55 ± 87.39
Previous botulinum toxin treatment, *n* (%)	23 (79.3)
Mean ± SD JRS severity score	3.24 ± 0.58
JRS severity score, *n* (%)	
1	0 (0)
2	2 (6.9)
3	18 (62.1)
4	9 (31.0)
Mean ± SD JRS frequency score	2.72 ± 0.75
Mean ± SD JRS total score	5.97 ± 1.21
Mean ± SD BSDI score (*n* = 17)	1.65 ± 0.90
Patients with a medical history, *n* (%)	6 (20.7)
Patients with comorbidities, *n* (%)	27 (93.1)

Medical history refers to any past medical or surgical history relevant to patient inclusion or exclusion criteria, as well as any past medical history deemed necessary for discussing the efficacy and safety of incobotulinumtoxinA. BMI, body mass index; BSDI, Blepharospasm Disability Index; FAS, full analysis set; JRS, Jankovic Rating Scale; SD, standard deviation.

**Table 2:** Change from baseline to 6 weeks after the first injection in the JRS severity score (MMRM analysis, FAS).

Change from Baseline in the JRS Severity Score	FAS (MMRM Analysis, *n* = 28)
LS mean ± SE	−2.08 ± 0.203
95% CI	−2.49, −1.66

The analysis for 6 weeks after the first injection was performed with 28 patients, and 1 patient who missed the scheduled visit was excluded from the analysis. CI, confidence interval; FAS, full analysis set; JRS, Jankovic Rating Scale; LS, least squares; MMRM, mixed-effects model for repeated measures; SE, standard error.

**Table 3:** Summary of AEs (safety population).

Patients with AEs	Safety Population (*n* = 29)
Any AEs	19 (65.5)
Any treatment-related AEs	7 (24.1)
AEs leading to death	0 (0.0)
Serious AEs	0 (0.0)
AEs leading to study discontinuation	0 (0.0)
AEs observed in ≥2 patients at the MedDRA PT level	
Nasopharyngitis	5 (17.2)
Eyelid ptosis	4 (13.8)
COVID-19	3 (10.3)
Vision blurred	2 (6.9)
Cough	2 (6.9)
Treatment-related AEs observed in ≥2 patients at the MedDRA PT level	
Eyelid ptosis	4 (13.8)

Results are presented as *n* (%). AE, adverse event; COVID-19, coronavirus disease 2019; MedDRA, Medical Dictionary for Regulatory Activities; PT, preferred term.

## Data Availability

Individual participant data underlying the results reported in this study are not publicly available. These data are owned by Teijin Pharma and are subject to legal and contractual restrictions.

## Supplementary Materials

The following supporting information can be downloaded at: https://www.mdpi.com/article/10.3390/toxins18020109/s1, Figure S1: Total number of administration sites for the first injection cycle in 29 patients (FAS); Figure S2: Scores in the fast blinking test (FAS); Figure S3: Graphical plain language summary (English); Figure S4: Graphical plain language summary (Japanese); Table S1: Summary of the last 2 botulinum toxin doses (safety population); Table S2: IncobotulinumtoxinA dose stratified by history of botulinum toxin treatment (FAS); Table S3: Dosing interval for incobotulinumtoxinA (FAS); Table S4: Dose and number of the first injection cycle sites stratified by affected muscle (FAS); Table S5: PEGR score at each injection cycle (FAS); Table S6: Summary of AEs by injection cycle (safety population); Table S7: Summary of AEs of special interest by injection cycle (safety population); Table S8: Supplementary methods.

## Abbreviations

The following abbreviations are used in this manuscript:

AE	Adverse event
BMI	Body mass index
BSDI	Blepharospasm Disability Index
CI	Confidence interval
COVID-19	Coronavirus disease 2019
EoT	End of trial
FAS	Full analysis set
JRS	Jankovic Rating Scale
LS	Least squares
MedDRA	Medical Dictionary for Regulatory Activities
MMRM	Mixed-effects model for repeated measures
PEGR	Patient Evaluation of Global Response
PT	Preferred term
SD	Standard deviation
SE	Standard error
SOC	System organ class
U	Unit
