# Supplementary material for: Efficacy and Safety of IncobotulinumtoxinA for the Treatment of Blepharospasm: A Multicenter, Phase 3 Study in Japan
Source: Toxins (Basel). 2026 Feb 20;18(2):109. doi: 10.3390/toxins18020109 (PMC12945157; doi:10.3390/toxins18020109)
Supplement: Supplementary file 1 [file toxins-18-00109-s001.zip › Figure S3.pdf]

Efficacy and Safety of IncobotulinumtoxinA for the Treatment of Blepharospasm: A Multicenter, Phase 3 Study in Japan

What is blepharospasm?

Blepharospasm is a condition in which the muscles around the eyes contract involuntarily, causing the eyes to close or become difficult to open regardless of your will. This condition commonly causes increased blinking, photophobia, dry eyes, and ocular pain.

What was the main purpose of the study?

The purpose of this study was to find out to what extent a treatment called incobotulinumtoxinA could improve the symptoms of blepharospasm and whether it can be safely administered to Japanese patients with blepharospasm.

How was this study performed?

- This study was conducted at 14 hospitals and clinics across Japan.
- 29 Japanese patients with blepharospasm (3 men and 26 women)
- Age: 18 to 80 years (average age: 64.6 years)
- Average Jankovic Rating Scale (JRS) score: 3.24 for severity, 2.72 for frequency, and 5.97 for total

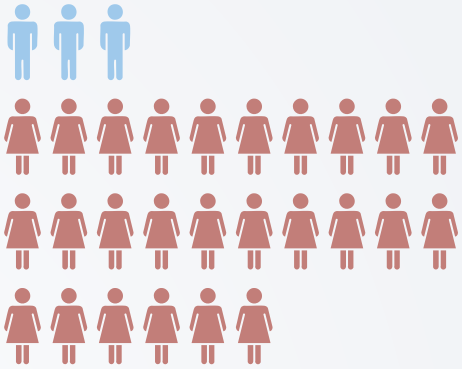

IncobotulinumtoxinA injections

<Dose>

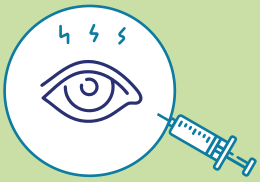

- 1st injection: 50, 75, or 100 units
- 2nd and later injections: Up to 100 units.

<Interval>

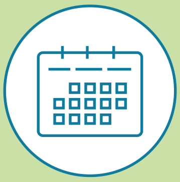

- Treatment interval: At least 6 weeks

The dose and dosing interval were determined by the trial doctor based on the blepharospasm symptoms.

Patients were followed up for 48 weeks, and the trial doctors used standard clinical scales (JRS and fast blinking test) and patient questionnaires, e.g., Blepharospasm Disability Index (BSDI), to check for changes in blepharospasm. Safety was checked throughout the study period.

What is the JRS score?

JRS is a clinical scale to measure the severity and frequency of blepharospasm symptoms on a 5-point scale. The total score was calculated by adding the severity and frequency scores. Higher scores mean more severe blepharospasm symptoms.

How was the fast blinking test performed?

Each patient was asked to blink for 10 seconds as quickly and lightly as possible. The trial doctor rated the patient’s blinking on a 4-point scale. Higher scores mean more difficulty in blinking.

What is the BSDI score?

BSDI is a functional scale to measure 6 daily living activities (reading, driving a vehicle, watching television, shopping, doing everyday activities, and walking). Each patient rated each of the activities on a 5-point scale. Higher scores mean more difficulty in daily living activities.

What were the main results of the study?

What were the main efficacy results?

Patients showed an improvement in blepharospasm (JRS severity score) after 6 weeks of the 1st injection. The observed change was considered clinically meaningful.
Repeated injections of incobotulinumtoxinA for 48 weeks resulted in sustained improvements in blepharospasm. In addition, patients felt improvements in self-assessed symptoms with repeated incobotulinumtoxinA injections.

Doctor's assessment

- Improvement in the JRS score (severity, frequency, and total)
- Improvement in the fast blinking test score

Patient's assessment

- Improvement in the BSDI score

What were the adverse events and treatment-related adverse events reported during the study?

All adverse events reported during the study were mild to moderate. During this study, the following adverse events and treatment-related adverse events were observed in 2 or more patients. There were no serious adverse events during the study. None of the patients stopped incobotulinumtoxinA treatment because of adverse events.

Adverse events (in 2 or more patients)

- Common cold (nasopharyngitis): **5 patients (17.2%)**
- Blurred vision (vision blurred): **2 patients (6.9%)**
- Drooping eyelid (blepharoptosis): **4 patients (13.8%)**
- Cough: **2 patients (6.9%)**
- COVID-19: **3 patients (10.3%)**

Treatment-related adverse events (in 2 or more patients)

- Drooping eyelid (blepharoptosis): **4 patients (13.8%)**

What is an adverse event, and what is a treatment-related adverse event?

An adverse event is any medical problem that a patient has during a trial. A treatment-related adverse event is an adverse event reported by the trial doctor as possibly related to trial treatment. An adverse event or treatment-related adverse event is considered serious when it is life-threatening, causes lasting problems, or requires hospital care.

How has this study helped patients and trial doctors?

This study showed that incobotulinumtoxinA injected into the muscles around the eyes was efficacious and well tolerated in Japanese patients with blepharospasm.

Where can I learn more about this study?

You can find more information about this trial on the following website: https://jrct.mhlw.go.jp/en-latest-detail/jRCT2031230711
Full study title: An open-label, uncontrolled, single-arm study of NT 201 in patients with blepharospasm
Clinical trial ID: jRCT2031230711
Trial sponsor: Teijin Pharma Limited
