# Supplementary material for: Efficacy and Safety of IncobotulinumtoxinA for the Treatment of Blepharospasm: A Multicenter, Phase 3 Study in Japan
Source: Toxins (Basel). 2026 Feb 20;18(2):109. doi: 10.3390/toxins18020109 (PMC12945157; doi:10.3390/toxins18020109)
Supplement: Supplementary file 1 [file toxins-18-00109-s001.zip › Figure S4.pdf]

眼瞼痙攣の治療におけるインコボツリヌストキシンAの有効性および安全性：国内多施設共同第3相試験

眼瞼痙攣とはどんな病気ですか？

眼瞼痙攣（がんけんけいれん）は、目のまわりの筋肉が異常に収縮し、勝手に目を閉じてしまう、または目が開けにくくなってしまう病気です。
眼瞼痙攣は、まばたきの増加、光過敏、ドライアイ、目の痛みなどを引き起こします。

この治験の目的は何ですか？

日本人の眼瞼痙攣の患者さんに対して、インコボツリヌストキシンAという治験薬を使用しました。
眼瞼痙攣の症状がどれくらい改善できるかという治験薬の効果（有効性）と、治験薬の安全性を評価しました。

この治験はどのように行われましたか？

- この治験は、日本全国の14の病院およびクリニックで行われました。
- 日本人の眼瞼痙攣の患者さん29名（男性3名、女性26名）
- 年齢18歳～80歳（平均年齢64.6歳）
- 平均のJankovic評価スケール（JRS）スコア：重症度3.24、頻度2.72、合計5.97

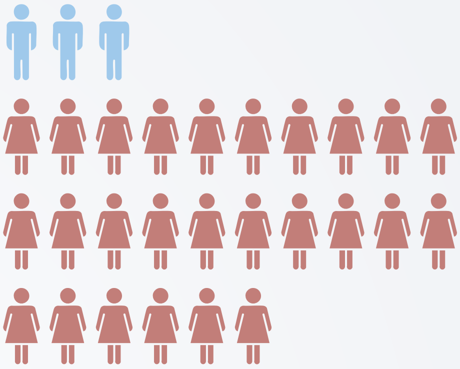

インコボツリヌストキシンAの注射方法

<注射量>

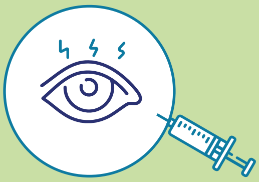

- 1回目：50、75、100単位のいずれか
- 2回目以降：100単位を超えないように設定

<注射間隔>

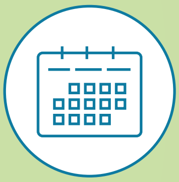

- 少なくとも6週間以上あけて注射

注射する量や注射間隔は、患者さんそれぞれの眼瞼痙攣の症状に応じて、医師が決めました。

患者さんは、48週間にわたって治験に参加しました。
医師は、JRSスコアや速瞬テスト、質問票への患者さんの回答（BSDI）などを用いて、眼瞼痙攣の症状の変化を確認しました。また、安全性を確認しました。

JRSスコアとは何ですか？

患者さんの眼瞼痙攣の「症状の重さ（重症度）」と「症状の起こる頻度」を医師が評価する方法です。
眼瞼痙攣の重症度と頻度をそれぞれ5段階で評価します。
合計スコアは、重症度のスコアと頻度のスコアを足した値です。
スコアが高いほど、眼瞼痙攣の症状が悪いことを示します。

速瞬テストとは、何ですか？

患者さんができるだけ速く軽いまばたきを10秒間行い、それを医師が見て、患者さんのまばたきを4段階で評価する方法です。
スコアが高いほど、まばたきがより難しいことを示します。

BSDIスコアとは何ですか？

質問票を使って、日常生活の6つの活動（読書、車の運転、テレビをみる、買い物、日常活動、歩行）の程度を患者さんが回答して評価する方法です。
6つの活動のそれぞれについて、5段階で評価します。
スコアが高いほど、日常生活活動が難しいことを示します。

この治験の主な結果は、どのようなものでしたか？

有効性の主な結果は、どうでしたか？

インコボツリヌストキシンAの1回目の注射から6週間後の時点で、眼瞼痙攣の症状（JRS重症度スコア）が改善することがわかりました。

インコボツリヌストキシンAを繰り返し注射することによって、48週間にわたって眼瞼痙攣の症状の改善が続くことがわかりました。また、質問票を用いた患者さん本人の評価によっても、眼瞼痙攣の症状が改善することがわかりました。

医師による評価

- JRS重症度スコア（重症度、頻度、および合計）の改善
- 速瞬テストスコアの改善

患者さんによる評価

- BSDIスコアの改善

治験中に報告された有害事象と副作用はどのようなものでしたか？

この治験中に、重篤な有害事象の報告はありませんでした。
２名以上の報告があった重篤でない有害事象と副作用は以下の通りでした。
有害事象のためにこの治験を中止した患者さんはいませんでした。

2名以上の報告があった有害事象

- かぜ（上咽頭炎）：5名（17.2%）
- まぶたが下がる（眼瞼下垂）：4名（13.8%）
- 新型コロナウイルス感染症（COVID-19）：3名（10.3%）
- 目がかすむ（霧視）：2名（6.9%）
- せき（咳嗽）：2名（6.9%）

2名以上の報告があった副作用

- まぶたが下がる（眼瞼下垂）：4名（13.8%）

有害事象とは何ですか？副作用とは何ですか？

治験期間中に参加者にみられた健康上の問題を、有害事象と言います。
有害事象のうち、治験を行った医師が「治験で受けた治療と関係がある」として報告したものを、副作用と言います。
有害事象および副作用は、生命に影響を及ぼす場合、問題を長い期間引き起こす場合や入院して治療することが必要な場合には、重篤な有害事象、重篤な副作用と言います。

患者さんや医師にとって、この治験はどのように役立ちましたか？

日本人の眼瞼痙攣の患者さんに対して、眼のまわりにインコボツリヌストキシンAを注射して治療することは有効であり、多くの患者さんが無理なく続けられることがわかりました。

この治験の詳細を知るにはどうすればよいですか？

この治験に関する詳しい情報はこちらからご確認いただけます。 https://jrct.mhlw.go.jp/latest-detail/jRCT2031230711

治験の標題：眼瞼痙攣患者を対象としたNT 201の非盲検・非対照・単群試験
登録番号：jRCT2031230711
治験依頼者：帝人ファーマ株式会社
