# Supplementary material for: Efficacy and Safety of IncobotulinumtoxinA for the Treatment of Blepharospasm: A Multicenter, Phase 3 Study in Japan
Source: Toxins (Basel). 2026 Feb 20;18(2):109. doi: 10.3390/toxins18020109 (PMC12945157; doi:10.3390/toxins18020109)
Supplement: Supplementary file 1 [file toxins-18-00109-s001.zip › toxins-4066742 Supplementary material.pdf]

Supplementary Materials

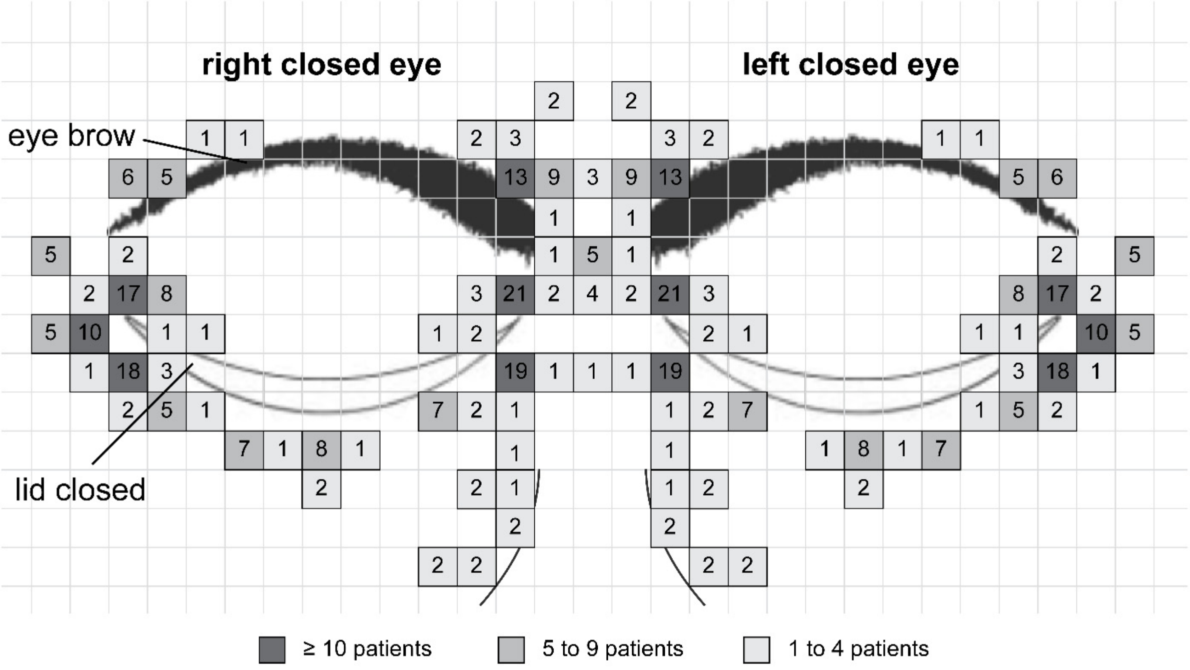

Figure S1. Total number of administration sites for the first injection cycle in 29 patients (FAS).

FAS, full analysis set.

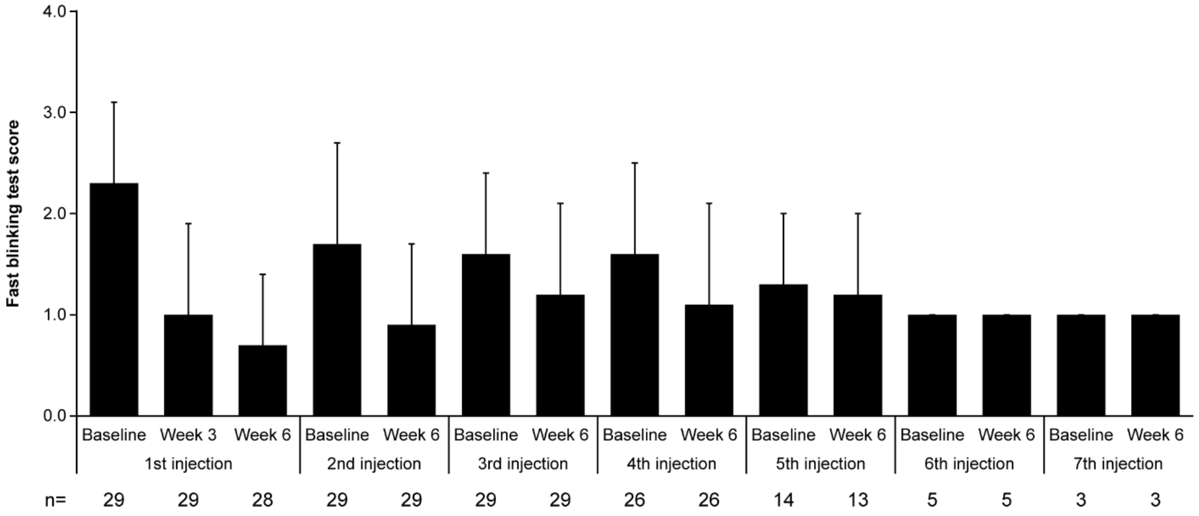

Figure S2. Scores in the fast blinking test (FAS).

Results are presented as mean and SD. The analysis for 6 weeks after the first injection was performed with 28 patients, and 1 patient who missed the scheduled visit was excluded from the analysis.

FAS, full analysis set; SD, standard deviation.

Table S1. Summary of the last 2 botulinum toxin doses (safety population).

	**Second-last injection cycle prior to trial entry (*n* = 22)**	**Most recent injection cycle prior to trial entry (*n* = 22)**
Total dose volume of onabotulinumtoxinA, U		
Mean ± SD	44.8 ± 17.502	45.98 ± 16.92
Median (range)	45.14 (17.5–100)	49 (21.3–100)
Total number of injection sites		
Mean ± SD	13.4 ± 3.2	13.4 ± 3.2
Median (range)	12.5 (9–21)	12.5 (9–21)
Injection interval (weeks)		
Mean ± SD	13.26 ± 3.475	17.02 ± 13.06
Median (range)	13 (8–21.7)	14.86 (9–73.1)

SD, standard deviation; U, unit.

Table S2. IncobotulinumtoxinA dose stratified by history of botulinum toxin treatment (FAS).

**IncobotulinumtoxinA dose**	**Injection cycle**						
	**1st (*n* = 29)**	**2nd (*n* = 29)**	**3rd (*n* = 29)**	**4th (*n* = 26)**	**5th (*n* = 14)**	**6th (*n* = 5)**	**7th (*n* = 3)**
All patients							
n	29	29	29	26	14	5	3
Mean ± SD dose, U	64.66 ± 18.319	68.17 ± 20.867	70.24 ± 24.229	70.65 ± 22.652	73.21 ± 20.719	80.00 ± 20.917	83.33 ± 28.868
Median (range) dose, U	50.00 (50.0–100.0)	75.00 (25.0–100.0)	75.00 (12.0–100.0)	75.00 (25.0–100.0)	75.00 (50.0–100.0)	75.00 (50.0–100.0)	100.00 (50.0–100.0)
≤50 U, *n* (%)	16 (55.2)	12 (41.4)	11 (37.9)	10 (38.5)	5 (35.7)	1 (20.0)	1 (33.3)
>50 to ≤75 U, *n* (%)	9 (31.0)	11 (37.9)	10 (34.5)	9 (34.6)	5 (35.7)	2 (40.0)	0 (0.0)
>75 to ≤100 U, *n* (%)	4 (13.8)	6 (20.7)	8 (27.6)	7 (26.9)	4 (28.6)	2 (40.0)	2 (66.7)

IncobotulinumtoxinA dose	Injection cycle						
	1st (*n* = 29)	2nd (*n* = 29)	3rd (*n* = 29)	4th (*n* = 26)	5th (*n* = 14)	6th (*n* = 5)	7th (*n* = 3)
Botulinum toxin-naïve patients							
n	6	6	6	5	2	0	0
Mean ± SD dose, U	50.00 ± 0.000	52.00 ± 12.410	47.83 ± 20.203	52.40 ± 13.831	50.00 ± 0.000	–	–
Median (range) dose, U	50.00 (50.0–50.0)	50.00 (37.0–75.0)	50.00 (12.0–75.0)	50.00 (37.0–75.0)	50.00 (50.0–50.0)	–	–
Patients who had previously received botulinum toxin treatment							
n	23	23	23	21	12	5	3
Mean ± SD dose, U	68.48 ± 18.795	72.39 ± 20.718	76.09 ± 21.948	75.00 ± 22.361	77.08 ± 19.824	80.00 ± 20.917	83.33 ± 28.868
Median (range) dose, U	75.00 (50.0–100.0)	75.00 (25.0–100.0)	75.00 (25.0–100.0)	75.00 (25.0–100.0)	75.00 (50.0–100.0)	75.00 (50.0–100.0)	100.00 (50.0–100.0)

FAS, full analysis set; SD, standard deviation; U, unit.

Table S3. Dosing interval for incobotulinumtoxinA (FAS).

Injection period	*n*	Dosing interval (weeks)				
		**<6**	**≥6 to <8**	**≥8 to <12**	**≥12 to <16**	**≥16**
Between 1st and 2nd injection cycles	29	0 (0.0)	5 (17.2)	10 (34.5)	10 (34.5)	4 (13.8)
Between 2nd and 3rd injection cycles	29	0 (0.0)	7 (24.1)	12 (41.4)	6 (20.7)	4 (13.8)
Between 3rd and 4th injection cycles	26	0 (0.0)	5 (19.2)	11 (42.3)	9 (34.6)	1 (3.8)
Between 4th and 5th injection cycles	14	0 (0.0)	2 (14.3)	11 (78.6)	1 (7.1)	0 (0.0)
Between 5th and 6th injection cycles	5	0 (0.0)	1 (20.0)	4 (80.0)	0 (0.0)	0 (0.0)
Between 6th and 7th injection cycles	3	0 (0.0)	3 (100.0)	0 (0.0)	0 (0.0)	0 (0.0)

Results are presented as *n* (%).

FAS, full analysis set.

Table S4. Dose and number of the first injection cycle sites stratified by affected muscle (FAS).

**Affected muscle**	***n***	**All patients (*n* = 29)**			**Botulinum toxin-naïve patients (*n* = 6)**			**Patients who had previously received botulinum toxin treatment (*n* = 23)**		
		**Total dose (U)**	**Dose per site (U)**	**Number of injection sites**	**Total dose (U)**	**Dose per site (U)**	**Number of injection sites**	**Total dose (U)**	**Dose per site (U)**	**Number of injection sites**
Orbicularis oculi	29	46.59 ± 19.426	4.48 ± 2.009	10.6 ± 1.3	31.25 ± 6.274	2.74 ± 0.382	11.3 ± 1.0	50.59 ± 19.759	4.94 ± 2.015	10.4 ± 1.3
Corrugator supercilii	27	11.02 ± 5.448	5.02 ± 1.744	2.2 ± 0.8	8.26 ± 2.843	4.41 ± 0.917	1.8 ± 0.4	11.81 ± 5.799	5.19 ± 1.898	2.3 ± 0.9
Procerus	10	4.73 ± 0.886	4.43 ± 0.917	1.1 ± 0.3	5.00 ± 0.000	5.00 ± 0.000	1.0 ± 0.0	4.62 ± 1.062	4.19 ± 1.016	1.1 ± 0.4

**Affected muscle**	***n***	**All patients (*n* = 29)**			**Botulinum toxin-naïve patients (*n* = 6)**			**Patients who had previously received botulinum toxin treatment (*n* = 23)**		
		**Total dose (U)**	**Dose per site (U)**	**Number of injection sites**	**Total dose (U)**	**Dose per site (U)**	**Number of injection sites**	**Total dose (U)**	**Dose per site (U)**	**Number of injection sites**
Nasalis	9	5.19 ± 0.747	2.59 ± 0.374	2.0 ± 0.0	5.00 ± 0.000	2.50 ± 0.000	2.0 ± 0.0	5.28 ± 0.929	2.64 ± 0.464	2.0 ± 0.0
LLSAN	1	5.00 ± 0.0	2.50 ± 0.0	2.0 ± 0.0	–	–	–	5.00 ± 0.0	2.50 ± 0.0	2.0 ± 0.0
Frontalis	14	9.09 ± 1.490	4.55 ± 0.745	2.0 ± 0.0	8.23 ± 2.052	4.11 ± 1.026	2.0 ± 0.0	9.44 ± 1.158	4.72 ± 0.579	2.0 ± 0.0

Results are presented as mean ± SD.

FAS, full analysis set; LLSAN, levator labii superioris alaeque nasi; SD, standard deviation; U, unit.

Table S5. PEGR score at each injection cycle (FAS).

**Time point**	***n***	**PEGR score**
1st injection cycle	29	2.0 ± 1.13
2nd injection cycle	29	1.7 ± 1.13
3rd injection cycle	29	1.3 ± 1.26
4th injection cycle	26	1.6 ± 1.06
5th injection cycle	14	1.3 ± 1.14
6th injection cycle	5	1.0 ± 1.58
7th injection cycle	3	2.3 ± 1.15

Results are presented as mean ± SD.

FAS, full analysis set; PEGR, Patient Evaluation of Global Response; SD, standard deviation.

Table S6. Summary of AEs by injection cycle (safety population).

**Patients with AEs**	**Overall (*n* = 29)**	**Injection cycle**						
		**1st (*n* = 29)**	**2nd (*n* = 29)**	**3rd (*n* = 29)**	**4th (*n* = 26)**	**5th (*n* = 14)**	**6th (*n* = 5)**	**7th (*n* = 3)**
Any AEs	19 (65.5)	11 (37.9)	9 (31.0)	6 (20.7)	8 (30.8)	3 (21.4)	1 (20.0)	0 (0.0)
Any treatment-related AEs	7 (24.1)	6 (20.7)	1 (3.4)	1 (3.4)	1 (3.8)	0 (0.0)	0 (0.0)	0 (0.0)
AEs leading to death	0 (0.0)	0 (0.0)	0 (0.0)	0 (0.0)	0 (0.0)	0 (0.0)	0 (0.0)	0 (0.0)
Serious AEs	0 (0.0)	0 (0.0)	0 (0.0)	0 (0.0)	0 (0.0)	0 (0.0)	0 (0.0)	0 (0.0)
AEs leading to study discontinuation	0 (0.0)	0 (0.0)	0 (0.0)	0 (0.0)	0 (0.0)	0 (0.0)	0 (0.0)	0 (0.0)
AEs observed in ≥2 patients at the MedDRA PT level								
Nasopharyngitis	5 (17.2)	2 (6.9)	1 (3.4)	2 (6.9)	2 (7.7)	0 (0.0)	0 (0.0)	0 (0.0)
Eyelid ptosis	4 (13.8)	4 (13.8)	0 (0.0)	0 (0.0)	0 (0.0)	0 (0.0)	0 (0.0)	0 (0.0)
COVID-19	3 (10.3)	3 (10.3)	0 (0.0)	0 (0.0)	0 (0.0)	0 (0.0)	0 (0.0)	0 (0.0)
Vision blurred	2 (6.9)	0 (0.0)	0 (0.0)	1 (3.4)	1 (3.8)	1 (7.1)	0 (0.0)	0 (0.0)
Cough	2 (6.9)	0 (0.0)	1 (3.4)	0 (0.0)	1 (3.8)	0 (0.0)	0 (0.0)	0 (0.0)

Treatment-related AEs observed in ≥2 patients at the MedDRA PT level								
Eyelid ptosis	4 (13.8)	4 (13.8)	0 (0.0)	0 (0.0)	0 (0.0)	0 (0.0)	0 (0.0)	0 (0.0)

Results are presented as *n* (%).

AE, adverse event; COVID-19, coronavirus disease 2019; MedDRA, Medical Dictionary for Regulatory Activities; PT, preferred term.

Table S7. Summary of AEs of special interest by injection cycle (safety population).

**Patients with AEs**	**Overall**	**Injection cycle**						
	**(*n* = 29)**	**1st (*n* = 29)**	**2nd (*n* = 29)**	**3rd (*n* = 29)**	**4th (*n* = 26)**	**5th (*n* = 14)**	**6th (*n* = 5)**	**7th (*n* = 3)**
Effects on distal muscles	7 (24.1)	4 (13.8)	1 (3.4)	1 (3.4)	1 (3.8)	1 (7.1)	0 (0.0)	0 (0.0)
Diplopia	1 (3.4)	0 (0.0)	1 (3.4)	0 (0.0)	0 (0.0)	0 (0.0)	0 (0.0)	0 (0.0)
Eyelid ptosis	4 (13.8)	4 (13.8)	0 (0.0)	0 (0.0)	0 (0.0)	0 (0.0)	0 (0.0)	0 (0.0)
Hypotonia	1 (3.4)	1 (3.4)	0 (0.0)	0 (0.0)	0 (0.0)	0 (0.0)	0 (0.0)	0 (0.0)
Vision blurred	2 (6.9)	0 (0.0)	0 (0.0)	1 (3.4)	1 (3.8)	1 (7.1)	0 (0.0)	0 (0.0)
Hypersensitivity reaction	2 (6.9)	1 (3.4)	0 (0.0)	0 (0.0)	1 (3.8)	0 (0.0)	0 (0.0)	0 (0.0)
Eczema	1 (3.4)	0 (0.0)	0 (0.0)	0 (0.0)	1 (3.8)	0 (0.0)	0 (0.0)	0 (0.0)
Rash	1 (3.4)	1 (3.4)	0 (0.0)	0 (0.0)	0 (0.0)	0 (0.0)	0 (0.0)	0 (0.0)
Eye disorders	9 (31.0)	6 (20.7)	1 (3.4)	1 (3.4)	2 (7.7)	1 (7.1)	0 (0.0)	0 (0.0)
Diplopia	1 (3.4)	0 (0.0)	1 (3.4)	0 (0.0)	0 (0.0)	0 (0.0)	0 (0.0)	0 (0.0)
Dry eye	1 (3.4)	0 (0.0)	0 (0.0)	0 (0.0)	1 (3.8)	0 (0.0)	0 (0.0)	0 (0.0)
Eyelid ptosis	4 (13.8)	4 (13.8)	0 (0.0)	0 (0.0)	0 (0.0)	0 (0.0)	0 (0.0)	0 (0.0)

**Patients with AEs**	**Overall**	**Injection cycle**						
	**(*n* = 29)**	**1st**	**2nd**	**3rd**	**4th**	**5th**	**6th**	**7th**
		**(*n* = 29)**	**(*n* = 29)**	**(*n* = 29)**	**(*n* = 26)**	**(*n* = 14)**	**(*n* = 5)**	**(*n* = 3)**
Eye discharge	1 (3.4)	1 (3.4)	0 (0.0)	0 (0.0)	0 (0.0)	0 (0.0)	0 (0.0)	0 (0.0)
Lagophthalmos	1 (3.4)	1 (3.4)	0 (0.0)	0 (0.0)	0 (0.0)	0 (0.0)	0 (0.0)	0 (0.0)
Vision blurred	2 (6.9)	0 (0.0)	0 (0.0)	1 (3.4)	1 (3.8)	1 (7.1)	0 (0.0)	0 (0.0)
Conjunctival hyperaemia	1 (3.4)	1 (3.4)	0 (0.0)	0 (0.0)	0 (0.0)	0 (0.0)	0 (0.0)	0 (0.0)
Convulsive attacks	0 (0.0)	0 (0.0)	0 (0.0)	0 (0.0)	0 (0.0)	0 (0.0)	0 (0.0)	0 (0.0)
Injection site reaction	1 (3.4)	1 (3.4)	0 (0.0)	0 (0.0)	0 (0.0)	0 (0.0)	0 (0.0)	0 (0.0)
Injection site bruising	1 (3.4)	1 (3.4)	0 (0.0)	0 (0.0)	0 (0.0)	0 (0.0)	0 (0.0)	0 (0.0)

Results are presented as *n* (%).

AE, adverse event.

Table S8. Supplementary methods.

Item	Method details
Preparation of incobotulinumtoxinA injection	IncobotulinumtoxinA (Merz Therapeutics GmbH, Frankfurt am Main, Germany) was supplied in vials containing 100 units of lyophilized powder per vial. The lyophilized powder was reconstituted with saline immediately before injection. After reconstitution, the investigators were allowed to store the vial at 2–8°C, and the vial was used for injection within 24 h.
JRS	The JRS (contact information and permission to use: Mapi Research Trust, Lyon, France, https://eprovide.mapi-trust.org), a severity scale for blepharospasm [22,23], consists of 2 categories (severity and frequency), and each category is assessed on a 5-point scale (severity: 0 = none to 4 = severe; frequency: 0 = none to 4 = functionally blind due to persistent eye closure >50% of waking time) [20]. The total score is the sum of the severity and frequency scores and may range from 0 to 8 [20]. The JRS was administered by trained investigators at screening, first injection baseline, and 3 and 6 weeks after the first injection. From screening to 6 weeks after the first injection, the JRS assessment was performed by the same investigator and at the same timeframe and in the same room environment to eliminate potential biases and variability in results. The JRS was thereafter administered every 4 weeks from 10 weeks

	after the first injection to the initiation of the second injection and subsequently during the continuous injection cycles.
BSDI	The BSDI is a functional scale for daily living activities that consists of 6 items (reading, driving a vehicle, watching television, shopping, doing everyday activities, and walking). Each item is evaluated on a 5-point scale (0 = no impairment to 4 = not possible due to disease; patients can select “not applicable” when necessary) [19,20]. The BSDI score was calculated by dividing the sum of single item scores with responses other than “not applicable” by the number of single items with responses other than “not applicable.” The BSDI was self-administered by patients or their representatives at screening, first injection baseline, 3 and 6 weeks after the first injection, every 4 weeks from 10 weeks after the first injection to the initiation of the second injection, and subsequently during the continuous injection cycles.
PEGR	The PEGR is a self-administered scale developed for the assessment of botulinum toxin treatment and is assessed on a 9-point scale (−4 = Marked worsening in severity of pain and in function to +4 = Marked improvement in severity of pain and in function) [24]. The PEGR for each injection cycle was evaluated before the initiation of the next incobotulinumtoxinA injection by the patients or their representatives.

Fast blinking test	The fast blinking test [3] was performed in accordance with the Japanese guidelines for blepharospasm [5]. Each patient was asked to blink for 10 s as quickly and lightly as possible, and the results were rated on a 4-point scale (0 = I can blink lightly more than 30 times in 10 s in good rhythm, 1 = I can blink lightly about 30 times in 10 s with interruptions, 2 = I can't blink lightly in good rhythm; I sometimes blink tightly, or 3 = I can't blink quickly and lightly) [3]. The test was performed at first injection baseline, 3 and 6 weeks after the first injection, every 4 weeks from 10 weeks after the first injection to the initiation of the second injection, and subsequently during the continuous injection cycles.

BSDI, Blepharospasm Disability Index; JRS, Jankovic Rating Scale; PEGR, Patient Evaluation of Global Response.
